# Cytocompatibility of Aqueous Garlic Extract on Human Cell Lines: Implications for Sustainability Utilization

**DOI:** 10.3390/ijerph23081056

**Published:** 2026-08-14

**Authors:** Eva Masciarelli, Laura Casorri, Claudio Beni, Massimiliano Valentini, Marcella Reale, Erica Costantini

**Affiliations:** 1 Department of Technological Innovations and Safety of Plants, Products and Anthropic Settlements, National Institute for Insurance Against Accidents at Work, via R. Ferruzzi, 38/40, 00143 Rome, Italy; e.masciarelli@inail.it (E.M.); l.casorri@inail.it (L.C.); 2 Research Centre for Engineering and Agro-Food Processing, Council for Agricultural Research and Economics, via della Pascolare, 16, Monterotondo, 00015 Rome, Italy; claudio.beni@crea.gov.it; 3 Research Centre for Food and Nutrition, Council for Agricultural Research and Economics, via Ardeatina, 546, 00178 Rome, Italy; massimiliano.valentini@crea.gov.it; 4 Department Innovative Technologies in Medicine and Dentistry, University “G. d’Annunzio”, via dei Vestini, 31, 66100 Chieti, Italy; mreale@unich.it

**Keywords:** aqueous garlic extract, farmers’ safety, human cell lines, in vitro tests, pesticides

## Abstract

**Highlights:**

**Public health relevance—How does this work relate to a public health issue?**

- The use of pesticides in agriculture poses health risks to agricultural workers and consumers. Natural remedies, such as garlic extract, can be used as an alternative.
- This study investigates the potential cytotoxicity of garlic extract to determine whether it can be safely used by agricultural workers as an alternative to pesticides.

**Public health significance—Why is this work of significance to public health?**

- Previous studies have shown that garlic extract is an excellent alternative to pesticides from an agronomic perspective.
- This study also showed no significant cytotoxic effects observed at realistic exposure concentrations (≤1%). This is an important finding for the safety of agricultural workers and consumers.

**Public health implications—What are the key implications or messages for practitioners, policy makers and/or researchers in public health?**

- The results underline the need to find natural alternatives to pesticides in agriculture to safeguard workers’ and consumers’ health.
- These results could promote the development of standardized, safe, and effective formulations intended not only for sustainable agriculture but also for the production of nutraceuticals and functional agents capable of supporting the immune system.

**Abstract:**

Pesticides are widely recognized as hazardous chemicals with potential adverse effects on human health and the environment. Directive 2009/128/EC establishes a framework for the European Union to promote the sustainable use of pesticides and the adoption of alternative agricultural techniques. Specifically, aqueous extracts are complex mixtures of various chemical compounds, such as terpenes, alcohols, aldehydes, and phenols, that have potential herbicidal, insecticidal, and fungicidal properties. Therefore, they can be used as an alternative to pesticides. This study examined the cytocompatibility of 100, 10, 1 and 0.1% *Allium sativum* L. aqueous extract (ASE) on human cell lines. The aim was to evaluate its effects on viability and cell metabolic activity through in vitro testing to verify the absence of adverse effects for operators who, when using garlic extract in agriculture, may be exposed via dermal contact or inhalation. The results showed that 1 to 0.1% do not significantly affect cell proliferation or viability in all examined cell models, while higher concentrations (≥10%) induced cell line-dependent reductions in viability. In summary, the results suggest the potential use of garlic aqueous extract as a potential low-risk alternative to synthetic pesticides and support its safety for occupational exposure of agricultural workers.

## 1. Introduction

Improving crop productivity and quality has required ever-increasing use of pesticides in agriculture; although their use generates important social and economic benefits, the health risk for workers and consumers exposed to small but repeated doses of pesticides over time may be significantly increased [1]. Pesticides include a wide range of substances, of natural or synthetic origin, which can be classified according to their chemical nature, the type of action they perform, and the target organisms, including insecticides, fungicides, acaricides, herbicides, etc. It is well known that pesticides, due to their high intrinsic toxicity and non-biodegradability, are considered hazardous chemicals, harmful not only to human health but also to the maintenance of biodiversity and the structure of aquatic and terrestrial ecosystems [2,3]. Humans can be exposed directly to pesticides in the workplace or indirectly through environmental media, such as air, water, soil, and the food chain, which can be contaminated by pesticides [4,5,6]. The main routes of pesticide exposure are inhalation, skin contact, and oral exposure, causing both acute adverse effects and long-term effects following chronic exposure at low doses. Chronic exposure to pesticides can affect fundamental barrier tissue, such as skin epithelium, airways, and intestine, causing several adverse effects on human health, including poisoning; respiratory, reproductive, and neurological disorders; and immune-mediated chronic inflammatory diseases and chronic degenerative diseases, such as diabetes, cancer, Alzheimer’s disease, and Parkinson’s disease, among others [7,8].

If not managed properly, they pose serious risks (respiratory, dermatitis, acute or chronic intoxication, oncological diseases) due to frequent applications, contact with treated crops, failure to use Personal Protective Equipment (PPE), and the performance of manual and mechanical operations before the product’s degradation time has elapsed. Chemical risk varies depending on the toxicity and hazardous nature of the product’s intrinsic properties, the level and duration of exposure, the degree of absorption through the respiratory and oral tracts, skin, and mucous membranes, as well as the method and frequency of use. Furthermore, the use of PPE (coveralls, gloves, self-contained breathing apparatus, or masks with P3 filters or FFP3 facepieces and eye protection) during pesticide applications is often poorly tolerated by workers due to their bulk and additional weight, which leads to increased fatigue and difficulty moving [9]. This situation often leads workers to ignore the requirement to wear PPE, increasing exposure and therefore the risk of intoxication or developing an occupational disease. Therefore, identifying safer plant-derived alternatives is not only an agronomic objective but also a preventive public health priority. In accordance with Directive 2009/128/EC [10], the dissemination of organic methods based on the sustainable use of pesticides is one of the main objectives aimed at limiting the risks posed by pesticide use to the environment and human health. Many studies were conducted on the need to reduce pesticide use, focusing attention on the use of natural extracts that could allow the gradual replacement of pesticides with plant extracts and be particularly effective in controlling pests and diseases of agricultural plants, despite not exhibiting significant toxicity. Furthermore, as a combination of compounds, they exhibit greater activity than any single active ingredient, resulting in a more effective response [11,12].

In recent years, interest has grown in the application of alternative agricultural methods, such as plant extracts, which offer significant advantages in terms of sustainable agriculture and represent a valid alternative against weeds and pests for crop disease control [13,14]. Aqueous extracts are complex mixtures of various chemical compounds (terpenes, alcohols, aldehydes, phenols) that possess potential herbicidal, insecticidal, and fungicidal properties and exhibit greater activity than the individual active ingredients (synergy), resulting in a more effective response [15,16,17]. Garlic (*Allium sativum* L.) is a widely cultivated plant, known for its nutritional value and its insecticidal, bactericidal, fungicidal, nematicidal, and repellent properties. Since 2014, numerous field trials have been conducted [18,19] to study the biostimulatory and repellent effects of an aqueous extract of *Allium sativum* L. on zucchini plants (*Cucurbita pepo* L.) subjected to spontaneous attacks by pests and pathogens. Field trials demonstrated the extract’s efficacy in controlling weeds and preventing agricultural diseases, as well as its biostimulatory properties.

Garlic water extract contains a high concentration of vitamins, particularly ascorbic acid and pyridoxine, with high antioxidant activity [20,21,22], and several amino acids known for their biostimulant effects [23,24].

The antioxidant activity of polyphenolic components in plants is also well documented. Biostimulant effects on zucchini plants in terms of both vegetative and reproductive parameters were already proven and suggested that the biostimulant effect may depend on its protective effect against oxidative stress [19,23,24,25,26]. One study showed the correlation between antioxidant properties and the presence of phenolic compounds in *Allium sativum* L. [19,27,28]. However, the synergistic action of the mixture of compounds has a greater effect than pure allicin alone [29].

Aqueous *Allium sativum* L. extract can therefore improve agricultural sustainability by acting as a natural crop strengthener and growth promoter, while reducing reliance on synthetic chemicals and minimizing negative environmental impacts such as soil and water contamination. Furthermore, aqueous garlic extract contributes to natural pest control by repelling and suppressing a wide range of pests, thus helping farmers manage crop damage in an environmentally friendly manner, without harming beneficial organisms or the environment. Indeed, garlic attracts beneficial insects, bees, and other pollinators, helping improve the overall health and productivity of agricultural crops. Furthermore, thanks to its bioactive compounds, potential human health benefits have been reported due to antioxidant, anti-inflammatory, antibacterial, antifungal, and immunomodulatory activities, demonstrating its promising use in the prevention and management of various diseases, as well as a functional food or nutraceutical.

Thus, it represents a promising alternative to synthetic chemical pesticides, with benefits for the environment. Keeping in mind all the properties of garlic extract, the benefits and advantage of its use, it was deemed appropriate to conduct a cytotoxicity assessment of garlic extract on human cell cultures to verify its safety for agricultural workers.

Garlic extracts differ significantly in their chemical composition and biological properties because different solvents extract different compounds. The use of organic solvents such as acetone, ethyl acetate, hexane, heptane, dichloromethane, methanol, ethanol, tetrahydrofuran, acetonitrile, dimethylformamide, toluene, and dimethyl sulfoxide allows for high levels of extraction of important substances. However, these substances pose a health risk if ingested and inhaled, as well as causing skin irritation and possible damage to the central nervous system (CNS) and immune system [30,31].

Aqueous extract is rich in water-soluble compounds like allicin, S-allylcysteine (SAC), and various proteins, carbohydrates, and tannins. The use of water has numerous advantages, such as a positive environmental impact (water is non-flammable and non- toxic to humans and the environment, provides the possibility of clean processing, and prevents pollution), the use of simple equipment, no risks, simplification of process steps, and the possibility of application in the food, agriculture and pharmaceutical industries [32].

Aqueous garlic extracts appear to be particularly effective in controlling pests and diseases of agricultural plants, while exhibiting no significant toxicity [32].

Garlic is known for its stimulant (allylthiamid), bactericidal, and fungicidal (allicin, alliin) properties, meaning it can stimulate plant growth and promote self-defense against fungal and bacterial attacks [11].

The most important compound present in the fresh bulb is alliin, a cysteine sulfoxide and an unstable, non-protein amino acid that degrades within sixteen hours at 23 °C. This molecule has low water solubility (2%) and is rapidly degraded and destroyed by heat.

Allicin is produced by the interaction of alliin with the enzyme alliinase. It has strong antiseptic, antibiotic, and antifungal effects (effective against bacteria, fungi, and viruses) and represents garlic’s natural defense against pests. It is also a good fungicide against powdery mildew, downy mildew, and other fungal infections. Furthermore, garlic extract, and especially its allicin, is known for its strong antioxidant properties [33,34]. Antioxidant activity plays an important role in plant stress management, and there is a positive correlation between increased antioxidant enzyme activity and varying tolerances to abiotic stress [34].

In this context, the cytocompatibility assessment of candidate biopesticides, like the aqueous garlic extracts, is an essential first step before their broader use in agricultural settings.

To date, there is no universally established safe concentration for aqueous garlic extracts. Low cytotoxicity was reported in Sprague-Dawley Rats, and an in vitro study indicated low toxicity with a CC_50_ greater than 1000 µg/mL (1 mg/mL) [35,36].

Taking into account previous studies demonstrating that 1% concentration is generally used in open-field experiments and that the levels of chemical compounds appear optimal for spraying on plants, by using different cell line models representative of different types of human tissues and organs for in vitro evaluation of safety of use by agricultural operators, the preliminary evaluation was studied with 100–10–1–0.1% concentration of the aqueous extract [37].

## 2. Materials and Methods

### 2.1. Preparation of Allium sativum Aqueous Extract for Cell-Based Experiments

The fine powder of micronized *Allium sativum* L. used for both field and in vitro experiments was purchased from the company BIOKI and stored at room temperature, until used for the experiments. Briefly, 0.5 g of *Allium sativum* L. was suspended in 50 mL of distilled water and incubated for 48 h on orbital shake. The obtained suspension *Allium sativum* L. aqueous extract (ASE) was then centrifuged at 1000 rmp for 20 min, and the supernatant was collected and filtered using a Millipore 0.22 µm vacuum-driven filter (Millipore, Billerica, MA, USA). The resulting extract was sterilized by UV irradiation and used for the following experiments.

### 2.2. Chemical Characterization of Allium sativum Aqueous Extract

About 10 mg of ASE was placed in a standard 5 mm NMR tube and were dissolved by adding 0.600 mL of D2O (99.8% deuterated) containing 0.05% (*v*/*v*) TMS. NMR experiments were carried out at the University of Perugia by using a Bruker AVANCE 600 MHz spectrometer (Bruker Biospin Gmbh, Rheinstetten, Germany) equipped with a 5 mm smartprobe (600 MHz for ^1^H) with a z gradient coil. The deuterium signal of D2O was used for locking and shimming of the B0 magnetic field, and the spectra were recorded at room temperature (298 K). ^1^H-NMR and ^13^C-NMR spectra signals were referenced to the TMS signal. 1D- and 2D-NMR spectra were recorded according to standard conditions by using a pulse program already loaded on the instrument database. All pulse sequences contained a pre-saturation of the water signal to cancel out its strong signal. ^1^H spectral width was set equal to 12.0 ppm, accumulating 32 scans with an acquisition time of 2.93 s, each with 64 K data points. 13C spectra were recorded over a 220 ppm window, accumulating 256 scans. ^1^H-^1^H-TOCSY experiments were collected in the phase-sensitive mode using Time Proportional Phase Incrementation (TPPI) for quadrature detection in the direct dimension, with a 4807 Hz spectral width in both dimensions, 100 ms of spin-lock time, 2K data points in f2, and 1K increments in f1, each with 32 scans. The water signal was suppressed. ^1^H-^13^C-HMQC spectra were acquired in TPPI phase-sensitive mode, with a 4807 Hz spectral width in f2 dimension and a 15,083 Hz spectral width in f1. 1K data points in f2 and 256 increments in f1, each with 32 scans, were used. HMQC was preferred to HSQC since the latter is more sensible for the optimization of the acquisition parameters.

### 2.3. Cell Culture

For studying the cellular and molecular mechanisms underlying pathophysiological processes, cell cultures represent a fundamental experimental model. In this study, human cell lines representative of different tissues and exposure routes were used to evaluate the cytocompatibility of the ASE. Cells maintained under standard culture conditions (37 °C, 5% CO_2_, humidified atmosphere) were used to evaluate responses to specific ASE treatments. All the medium and reagents were supplied by Merck Life Science NV, Amsterdam, The Netherlands.

### 2.4. SH-SY5Y

The human neuroblastoma cell line SH-SY5Y (Cytion GmbH, Heidelberg, Germany) were cultured in Dulbecco’s Modified Eagle Medium (DMEM) supplemented with 10% fetal bovine serum (FBS), 100 U/mL penicillin and 100 µg/mL streptomycin in a humidified incubator at 37 °C in a 5% CO_2_. The maintenance of cells was carried out per trypsinization once or twice a week and sub-cultivation in complete medium in a 1:3 split ratio. A total of 5 × 10^4^ cells/well were plated in a 96-well plate for subsequent experiments. Experiments were repeated three times, with three biological replicates. The results of triplicate analysis of each sample were consistent. For each experiment, the cultures were carefully matched in terms of all conditions including cell passage number, batch of medium, etc.

### 2.5. HaCaT

The immortalized human keratinocyte cell line (HaCaT) (Cytion GmbH, Heidelberg, Germany) was cultured in DMEM containing 5.5 mmol/L glucose with 10% heat-inactivated FBS supplemented and 4 mmol/L L-glutamine at 37 °C, under 5% CO_2_ in an incubator. After cells grew to 80% of confluent, a subculture was prepared at a ratio of 1:10. For all experiments, freshly trypsinized cells were seeded at a density of 5 × 10^4^ cells/well and allowed to adhere prior to treatment with ASE. Experiments were repeated three times, with three biological replicates. The results of triplicate analysis of each sample were consistent. For each experiment, the cultures were carefully matched in terms of all conditions including cell passage number, batch of medium, etc.

### 2.6. THP-1-Derived Macrophages

THP-1 (Cytion GmbH, Heidelberg, Germany), the human monocytic leukemia cell line, was maintained in RPMI-1640 medium, containing 10% heat-inactivated fetal calf serum and 200 µg/mL of penicillin and streptomycin. Cells were plated at a density of 7 × 10^4^ cells/well of 96 well-plates and maintained at 37 °C, 5% CO_2_. For differentiation into macrophage-like cells, THP-1 cells were treated with 20 nM phorbol-12-myristate-13-acetate (PMA) for 48 h, resulting in adherence and morphological changes consistent with macrophages. Experiments were repeated three times, with three biological replicates. The results of triplicate analysis of each sample were consistent. For each experiment, the cultures were carefully matched in terms of all conditions including cell passage number, batch of medium, etc.

### 2.7. Caco-2

The human epithelial colorectal adenocarcinoma cell line, Caco-2 (Cytion GmbH, Heidelberg, Germany), was grown in DMEM, supplemented with 10% of FBS, 2 mM L-glutamine and 1% MEM Amino Acids solution. Cell cultures were routinely maintained at 37 °C in a humidified atmosphere of 5% CO_2_, 95% air, in filter screw caps of 75 cm^2^ (Corning Inc., New York, NY, USA). Subcultured cells were seeded in a concentration of 10^4^ cells/cm^2^, and the medium was changed every 3 days until 100% confluence, checked by phase-contrast microscopy (Leica Biosystems, Amsterdam, The Netherlands). Then, cells were trypsinized (Trypsin-EDTA 0.05–0.02%) and plated in a 96-well plate (5 × 10^4^ cells/well) and maintained for 21 days in complete medium, changed three times a week to differentiate completely. Cells were then serum-starved for 18 h to synchronize all cells to the same cell cycle phase and remove the impact that the cell cycle would have on the response to treatment. Experiments were repeated three times, with three biological replicates. The results of triplicate analysis of each sample were consistent. For each experiment, the cultures were carefully matched in terms of all conditions including cell passage number, batch of medium, etc.

### 2.8. A549

A549 (Cytion GmbH, Heidelberg, Germany), the human lung adenocarcinoma epithelial cell line, was kept in Ham’s F-12 Medium (F-12K) supplemented with 10% (*v*/*v*) FBS, 1% of penicillin–streptomycin and 1% fungizone at 37 °C in a humidified incubator with 5% CO_2_ until it reached 80–90% confluency. Once the required number of cells was reached, A549 cells were placed in 96-well plate at the concentration of 5 × 10^4^ cells/well. Experiments were repeated three times, with three biological replicates. The results of triplicate analysis of each sample were consistent. For each experiment, the cultures were carefully matched in terms of all conditions including cell passage number, batch of medium, etc.

### 2.9. Cell Treatments

HaCaT, SH-SY5Y, A549, THP-1 derived-macrophages and Caco-2 cells were treated with the 100, 10, 1 and 0.1% of ASE for 24 and 48 h after reaching confluence. Cells plated in presence of completed medium were employed as control.

### 2.10. MTT Assay

**Figure 1:**
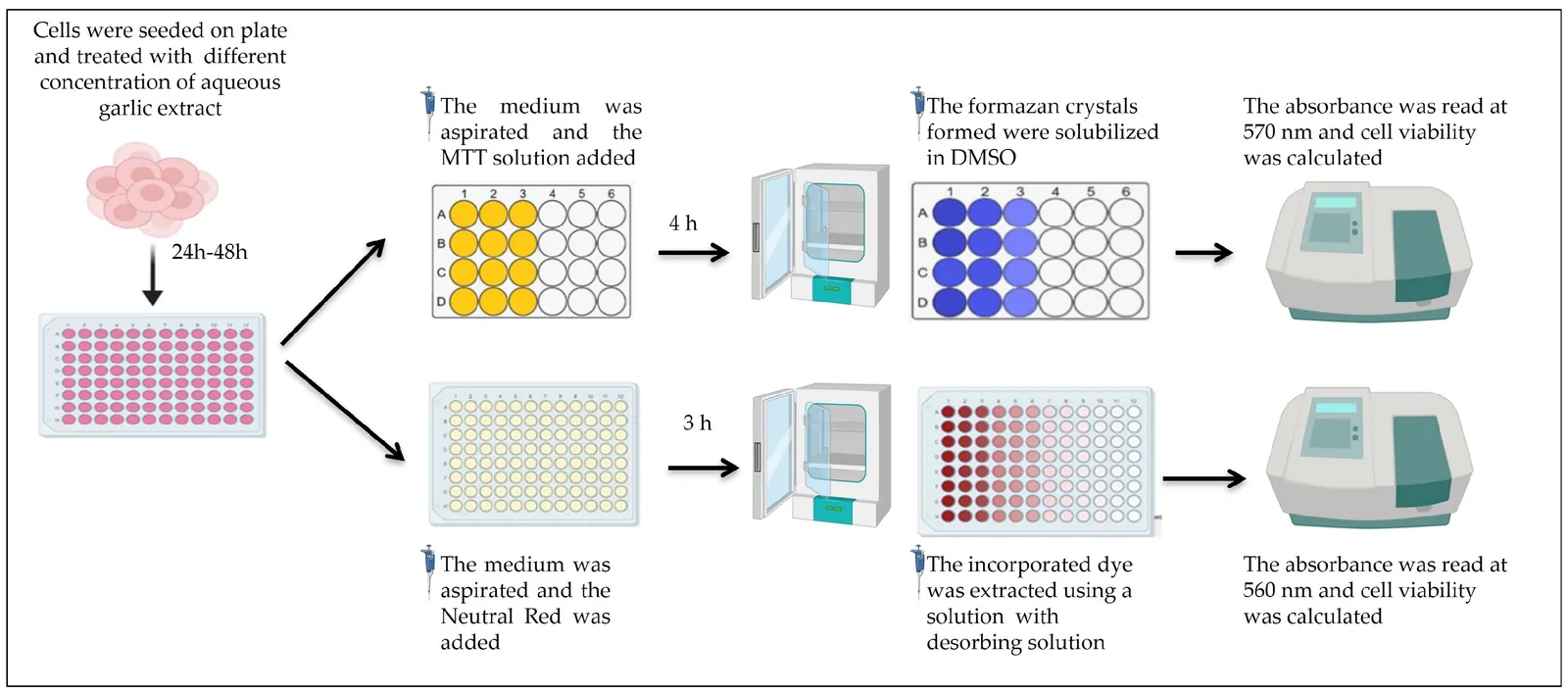
Representative image of viability assay procedures.

Cell viability was assessed after 24 h and 48 h ASE treatments using the MTT assay, which measures metabolic activity of mitochondria as an indicator of cell viability. Following treatment, the culture medium was removed and replaced with 100 μL of fresh medium containing 10 µL of MTT solution (5 mg/mL; MTT powder dissolved in PBS). Additional incubation at 37 °C, 5% CO_2_ was done for 4 h, then the medium was removed, and the resulting formazan crystals were dissolved in 100 μL of DMSO. The absorbance of formazan was measured at 570 nm using a microplate reader (Glomax Multidetection System, Promega, Italy) (Figure 1).

### 2.11. Neutral Red Uptake Assay

Cell viability was assessed also using the Neutral Red (NR) uptake assay, based on the ability of viable cells to incorporate and bind the supravital dye Neutral Red within lysosomes. Cells were seeded in 96-well plates at a density of 5 × 10^3^ cells/well and allowed to attach overnight under standard culture conditions (37 °C, 5% CO_2_). Following incubation, cells were treated with 100, 10, 1 and 0.1% of ASE for 24 h and 48 h. At the end of the treatment period, the culture medium was removed and replaced with fresh medium containing Neutral Red dye at a final concentration of 50 µg/mL. Cells were then incubated for 3 h at 37 °C to allow dye uptake. After incubation, the dye-containing medium was carefully aspirated, and cells were gently washed with phosphate-buffered saline (PBS) to remove unincorporated dye. Subsequently, the incorporated dye was extracted using a destaining solution composed of 50% ethanol, 49% distilled water, and 1% glacial acetic acid. Absorbance was measured at 560 nm using a microplate reader (Glomax Multidetection System, Promega, Italy). All experiments were performed in three independent replicates, each with three technical repeats. Data were analyzed using GraphPad Prism 9.0 software (Figure 1).

A qualitative method was used to evaluate the effect of different concentrations of ASE on cell cytocompatibility.

### 2.12. Statistical Analysis

All the data collected in this study were analyzed using GraphPad Prism 9.0 software. Normally distributed measurement data were expressed as mean  ±   standard error mean (SEM). When comparing parameters between concentrations, one-way ANOVA and Tukey’s post hoc test were applied. *p* < 0.05 was considered statistically significant.

## 3. Results

### 3.1. NMR Analysis of the Allium sativum Acqueous Extract

**Figure 2:**
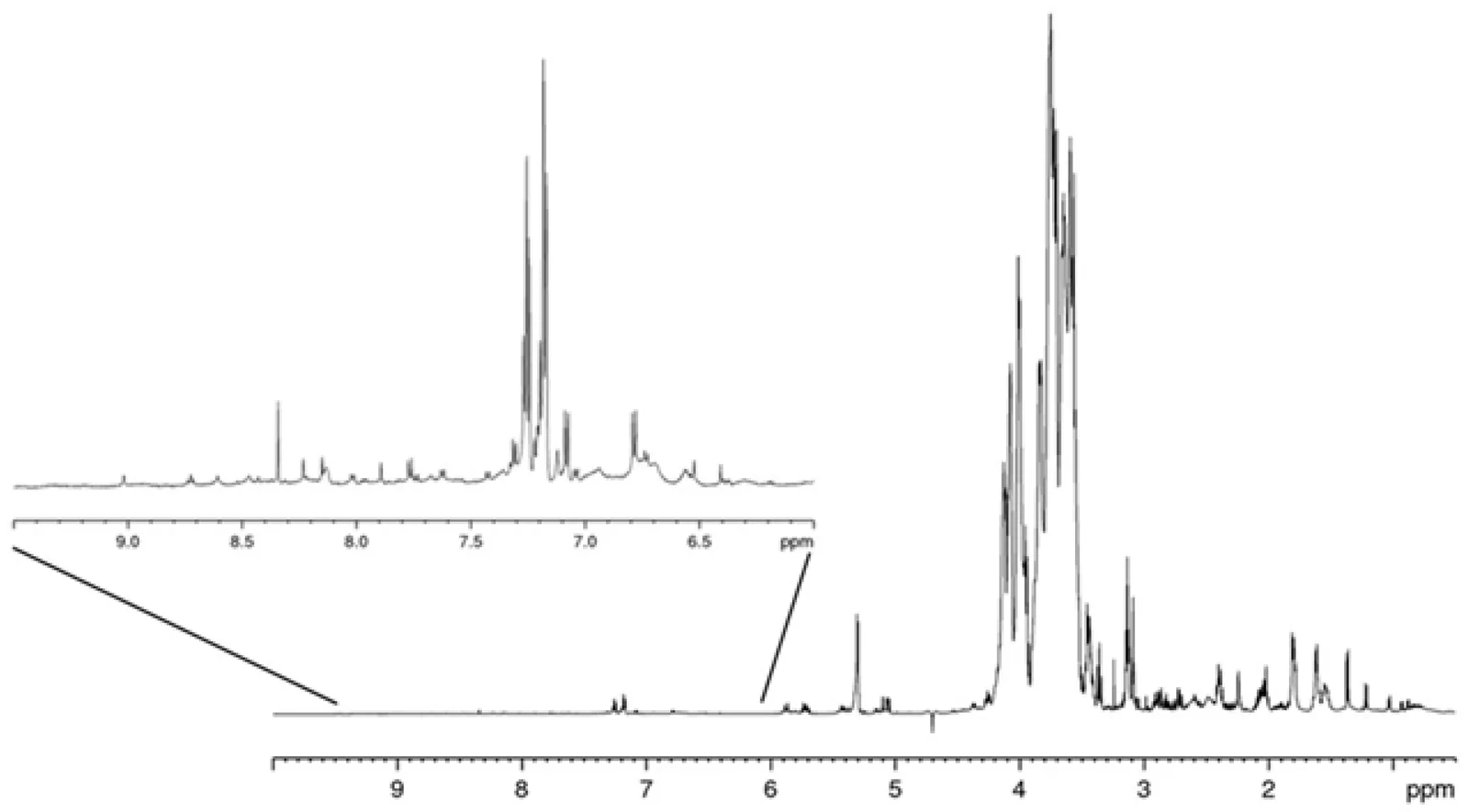
^1^H-NMR spectrum of ASE The enlargement on the upper left part refers to the region of the aromatic protons.

Figure 2 reports the ^1^H-NMR spectrum of the aqueous garlic extract from 0 to 10 ppm, while in the upper left part the expansion of the aromatic region is shown. The region around 1.5 ppm shows the presence of small traces of some fatty acids, such as saturated C18 and C16 acids (stearic and palmitic acids, respectively), as well as unsaturated fatty acids (C18:1, oleic acid, and C18:2, linoleic acid). The region between 3.00 and 4.50 ppm is the most intense of the spectrum and accounts for the presence of amino acids, such as asparagine, glutamine, arginine, threonine, etc., sugars, predominantly sucrose, and glucose. The region around 5.00 ppm shows the typical signals of allicin and probably allyl-organosulphur, which are present, if at all, in very small quantities. The aromatic region from 6.00 ppm and above accounts for the presence of traces of phenylalanine, tryptophan, tyrosine, formic and fumaric acid. The most relevant peaks are in the region between 5.00 and 6.50 ppm where the most abundant organosulphur compounds show the resonances of the double bonds. Despite this part of the NMR spectrum having several overlaps, it is possible to observe the presence of amounts of allicin and allyl-organosulphur molecules. The assignments of these important species agree with previous investigations [33].

### 3.2. In Vitro Effect of Allium sativum Acqueous Extract

Respiratory, skin, gastrointestinal, and circulating cells represent the first line of interaction between the organism and the external environment, playing a fundamental role in protection, communication, and maintaining homeostasis. Their functional integrity is essential for the health of tissues and the entire organism.

To assess the safety profile of *Allium sativum* aqueous extract, multiple human cell lines representative of different exposure routes were analyzed. Cytocompatibility was assessed by measuring mitochondrial metabolic activity through the MTT assay, and the lysosomes’ integrity and functionality was measured through the Neutral Red Assay, both after 24 and 48 h of exposure to increasing concentrations of the ASE (0.1–100%). The use of multiple human cell models strengthens the public health interpretation of the findings, as these models reflect tissues that may be relevant to occupational or environmental exposure: skin for dermal contact, respiratory epithelium for inhalation, intestinal epithelium for accidental ingestion or consumer exposure, immune cells for inflammatory response, and neuronal cells for potential neurotoxicity screening.

#### 3.2.1. Cytocompatibility of ASE on Caco-2 Cells

Gut barrier integrity is negatively affected by pesticide exposure with loss of villi, alteration junction and increase in permeability, dysbiosis, and inflammatory response. Thus, the presence of pesticides in our diet and persistent environmental exposure represents an important risk factor for developing chronic diseases [38,39]. Caco-2 cells were used as an in vitro model for the human small intestine, especially in interactions with food/drug components, drug discovery, drug absorption, permeability, transport mechanisms, and toxicity. The Caco-2 cell model has been widely used to assess the ability of chemicals to cross the intestinal barrier, mimicking intestinal function well after differentiating into polarized enterocyte-like cells, making them great for assessing how substances move across the gut barrier, evaluating formulations, and understanding cellular processes.

**Figure 3:**
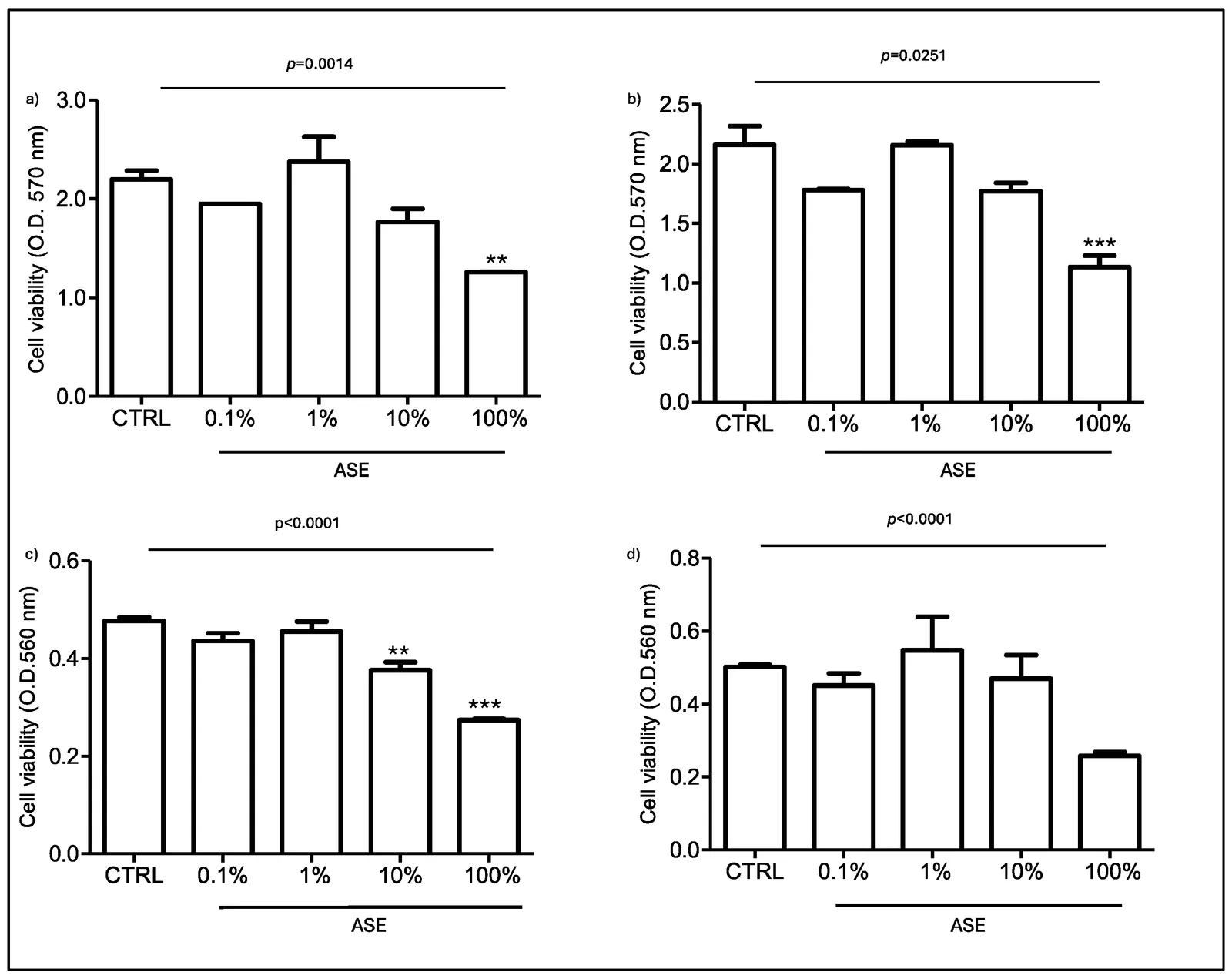
Caco-2 cell viability: Caco-2 cell viability was assessed by MTT (**a**,**b**) and NR (**c**,**d**) assays after 24 and 48 h of incubation with four different concentrations of ASE (100, 10, 1 and 0.1%). In the figure, (**a**,**c**) refer to 24 h, while (**b**,**d**) refer to 48 h. Data are expressed as mean ± SEM (n = 3). A one-way ANOVA was performed to assess overall differences between groups. Specific differences between each group and the control were determined using Tukey’s post hoc test. Asterisks indicate statistical significance levels compared to the control (** *p* < 0.01; *** *p* < 0.001).

The MTT assay showed a non-significant variation of cells at lower concentrations (0.1% and 1%) at both early and later time points of incubation. At 10%, a moderate and non-significant reduction in viability was observed (19.46% at 24 h and 17.94% at 48 h), while a significant decrease in viability was evident at 100% (42.71% at 24 h and 47.45% at 48 h), suggesting reduced cytocompatibility. A similar trend was observed in the NR assay (Figure 3). In fact, low concentrations of 0.1 and 1% do not affect cell viability after 24 or 48 h of incubation. However, at 10% a reduction in viability was detected after both 24 and 48 h (21.17% and 6.29%, respectively), demonstrating that cell viability is always higher than 75%, that is, the threshold for significant cytotoxicity. Only the highest concentration (100%) significantly affects Caco-2 viability, with a reduction at 42.55% and 48.58% after 24 h and 48 h of incubation, respectively.

Overall, both assays showed consistent results, with similar trends at 24 and 48 h and a significant reduction in cell viability observed only at the 100% concentration.

#### 3.2.2. Cytocompatibility of ASE Aque on SH-SY5Y Cells

Since a relationship between Parkinson’s disease and occupational exposure to pesticides has been repeatedly suggested [40], our objective was to assess the cytocompatibility of ASE on SH-SY5Y cells. The SH-SY5Y cell line, derived from human neuroblastoma, is extensively utilized in medical research as a relevant cell model to evaluate cellular neurotoxicity, to elucidate cellular and molecular impacts of neurotoxicants as organic environmental pollutants, to facilitate prioritization of in vivo testing, and to screen and validate drugs for their therapeutic effect against neurodegenerative diseases.

SH-SY5Y cell viability was evaluated by MTT and NR assays after 24 and 48 h of incubation with ASE. Results of the MTT assay showed that cell viability was similar to control levels at 24 h (mean values close to 100%), indicating no evident cytotoxic effect at this time point. In contrast, a marked reduction in viability was observed in cells incubated for 48 h in presence of ASE 10 and 100%, suggesting a time-dependent cytotoxic effect under these conditions.

**Figure 4:**
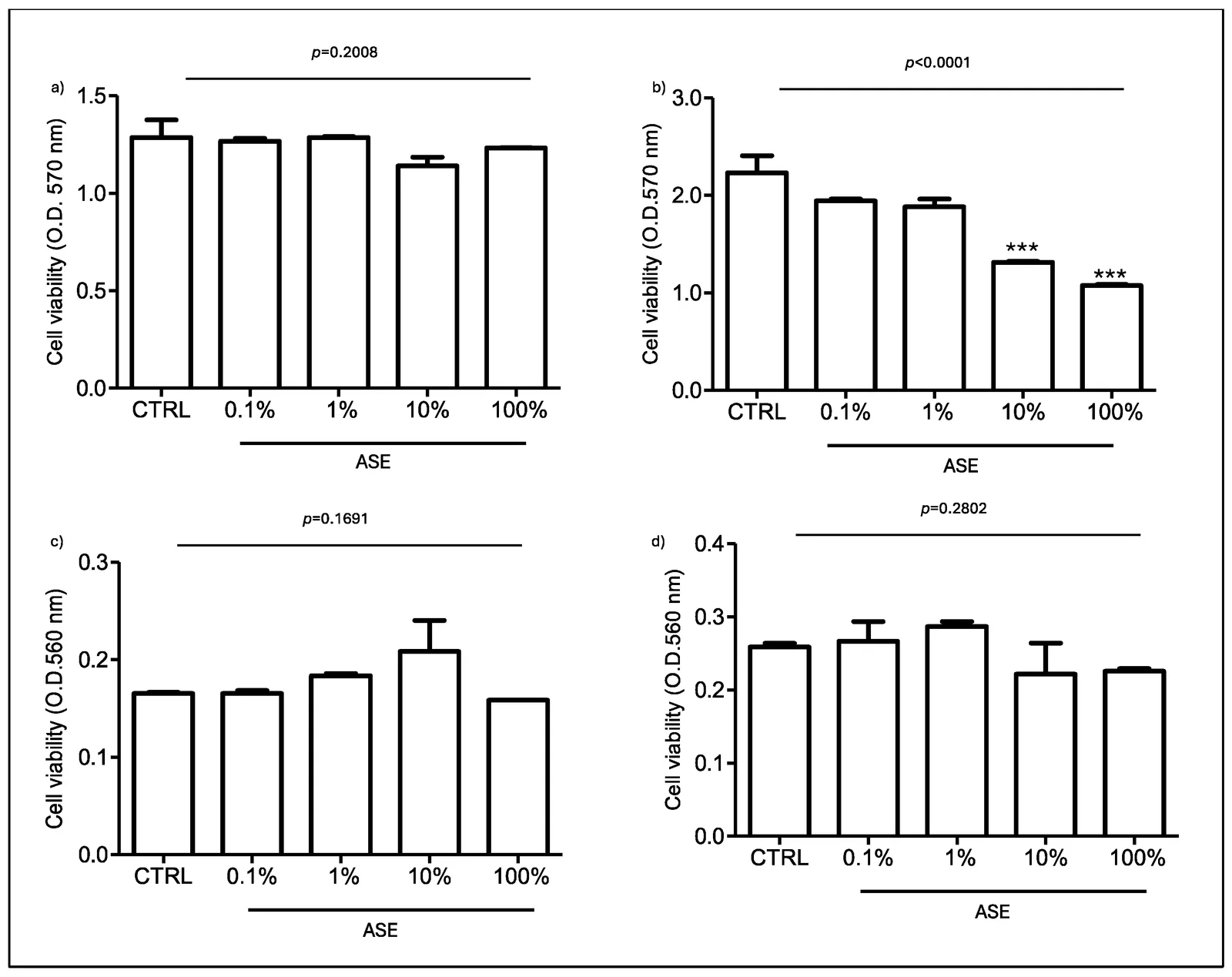
SHSY-5Y cell viability: SHSY-5Y cell viability was assessed by MTT (**a**,**b**) and NR (**c**,**d**) assays after 24 and 48 h of incubation with four different concentrations of ASE (100, 10, 1 and 0.1%). In the figure, (**a**,**c**) refer to 24 h, while (**b**,**d**) refer to 48 h. Data are expressed as mean ± SEM (n = 3). A one-way ANOVA was performed to assess overall differences between groups. Specific differences between each group and the control were determined using Tukey’s post hoc test. Asterisks indicate statistical significance levels compared to the control (*** *p* < 0.001).

In contrast, the NR assay does not show statistically significant variations at both 24 and 48 h (Figure 4).

#### 3.2.3. Cytocompatibility of Aqueous Garlic Extract on A549 Cells

**Figure 5:**
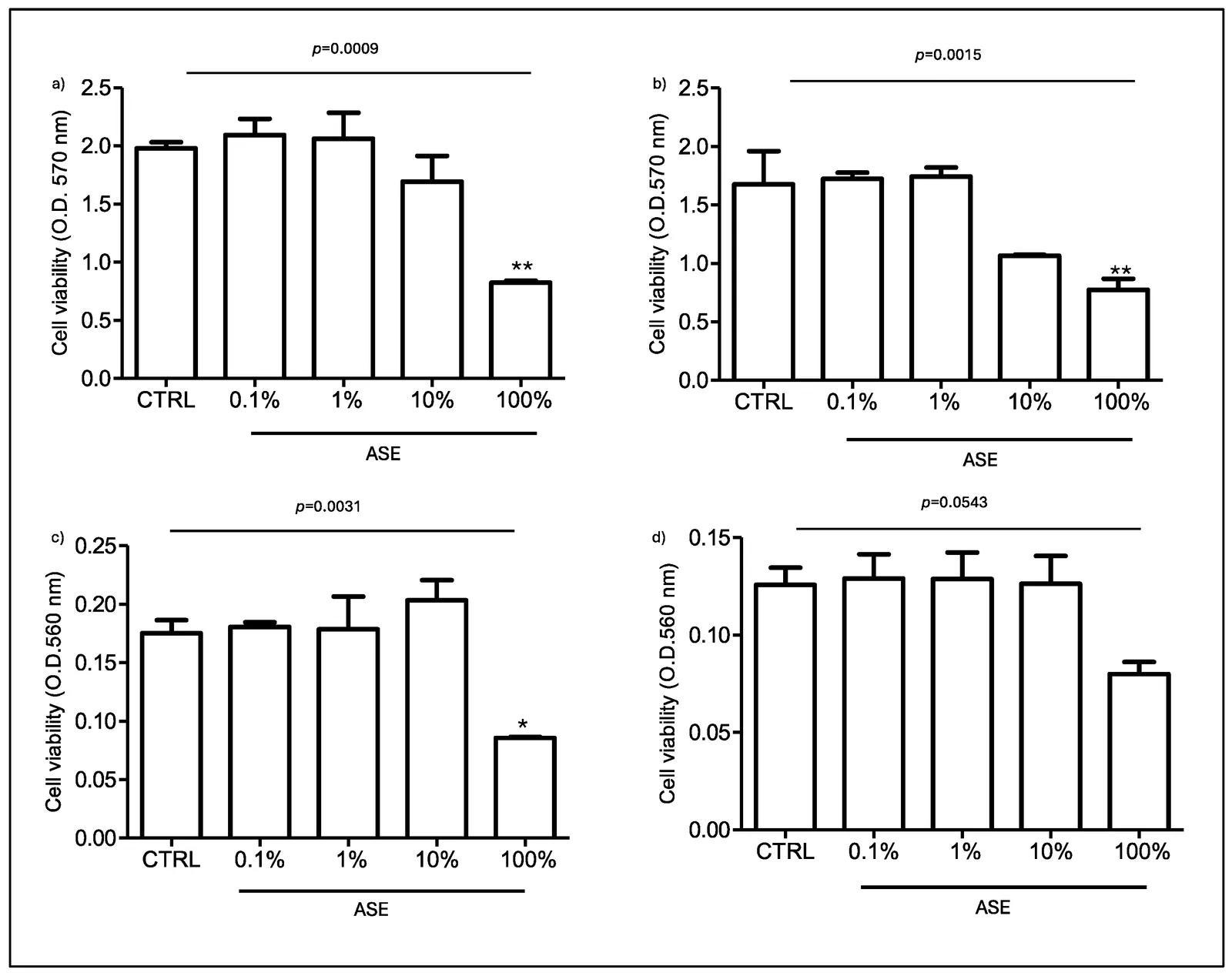
A549 cell viability: A549 cell viability was assessed by MTT (**a**,**b**) and NR (**c**,**d**) assays after 24 and 48 h of incubation with four different concentrations of ASE (100, 10, 1 and 0.1%). In the figure, (**a**,**c**) refer to 24 h, while (**b**,**d**) refer to 48 h. Data are expressed as mean ± SEM (n = 3). A one-way ANOVA was performed to assess overall differences between groups. Specific differences between each group and the control were determined using Tukey’s post hoc test. Asterisks indicate statistical significance levels compared to the control (* *p* < 0.05; ** *p* < 0.01).

Asthma, airway inflammation and bronchial hyperreactivity are increased prevalently in countries where the use of pesticides has intensified [41,42]. The A549 cell line, derived from human lung cancer, serves as a versatile model for studying type II pneumocytes to understand respiratory disease risk, the effects of airborne pollutants on the lungs, lung cancer, and respiratory infections, acting as a proxy for normal lung tissue and disease state. As reported in Figure 5, in the MTT assay, cell viability remained comparable to control values at the lowest concentrations (0.1% and 1%) at both 24 and 48 h, with values slightly above 100%. A moderate reduction was observed at 10%, with viability decreasing to 14.44% after 24 h and to 36.49% after 48 h. At 100%, a marked decrease in viability was detected, reaching 41.62% of cell viability at 24 h and 46.11% at 48 h, indicating reduced cytocompatibility of A549 with the highest concentration of ASE.

The NR assay showed a similar trend, with cell viability not influenced by low concentration of 1% and 10% of ASE. Only at 100% was a clear reduction in viability observed (Figure 5).

#### 3.2.4. Cytocompatibility of ASE on HaCaT Cells

**Figure 6:**
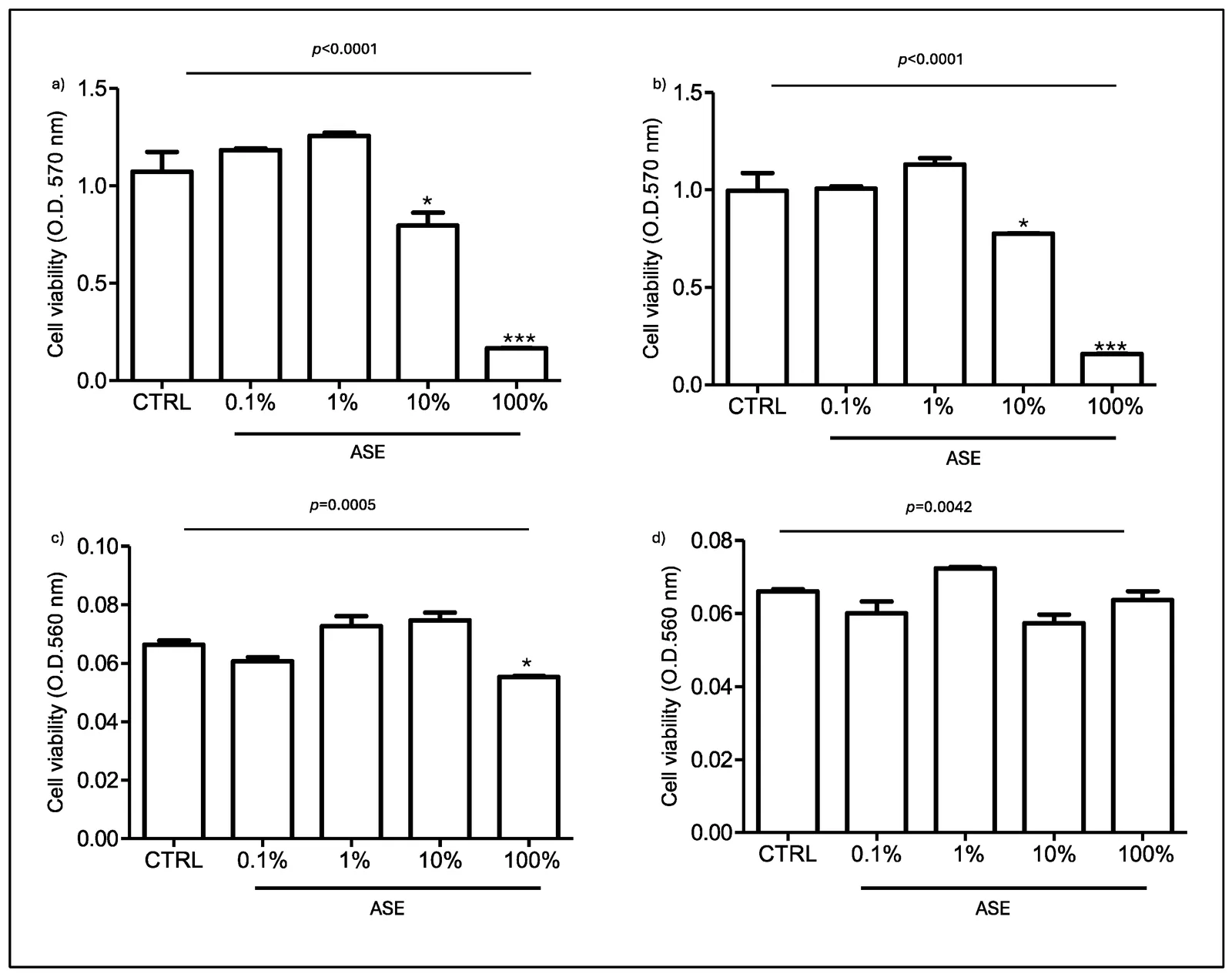
HaCaT cell viability: HaCaT cell viability was assessed by MTT (**a**,**b**) and NR (**c**,**d**) assays after 24 and 48 h of incubation with four different concentrations of ASE (100, 10, 1 and 0.1%). In the figure, (**a**,**c**) refer to 24 h, while (**b**,**d**) refer to 48 h. Data are expressed as mean ± SEM (n = 3). A one-way ANOVA was performed to assess overall differences between groups. Specific differences between each group and the control were determined using Tukey’s post hoc test. Asterisks indicate statistical significance levels compared to the control (* *p* < 0.05; *** *p* < 0.001).

The skin represents a possible and common route of entry for chronic exposure to pesticides that can be absorbed through the epidermal layers of the body, and the skin itself is a target organ of pesticide toxicity [43]. HaCaT cells, from adult human skin, are an immortalized human keratinocyte line that exhibits the functional characteristics of primary cells, forms stratified layers, and reacts in a predictable manner to stimuli. HaCaT cells are useful in modeling skin barrier development, UV response, oxidative stress response, cosmetics, and topicals. Their stability and physiologically appropriate behavior enabled the researchers to simulate wound healing, barrier function and inflammatory processes. For this reason, in this preliminary study we used HaCaT cells to evaluate the cytocompatibility of garlic extract. The results of the MTT assay showed non-statistically significant variation in cell viability at the lowest concentrations (0.1% and 1%) at both time points. A statistically significant reduction was observed at 10 and 100% at both 24 and 48 h. With ASE 10%, reductions in viability were moderate, about 25.80% and 22.09%, respectively, at 24 and 48 h. The highest concentration (100%) caused a statistically significant reduction in cell viability (Figure 6). Also, the NR assay showed no significant effect of 0.1 and 1% ASE on HaCaT cell viability. Only at 100% did ASE induce a reduction in viability after 24 h, detected by the NR assay.

#### 3.2.5. Cytocompatibility of Aqueous Garlic Extract on THP-1-Derived Macrophages

Prolonged or high-level exposure, especially in agricultural workers, has been linked to alteration of immune functions, increasing infection risk or allergies and autoimmune diseases [44]. Due to its potential effects on the immune system, we investigated the interaction of garlic extract with macrophages, important cells of the immune system involved in innate and specific immune responses. After differentiating them into macrophage-like cells, human monocytic cell line THP-1 cells were used for studying monocyte/macrophage biology, immune responses, inflammation, and lipid metabolism; as models for cancer/infectious diseases; and for drug screening, cytokine studies, and co-culture systems, mimicking real immune cell functions in vitro.

Both assays showed a relatively mild concentration-dependent reduction in cell viability, with limited effects at lower concentrations and a more evident decrease at the highest concentration tested.

**Figure 7:**
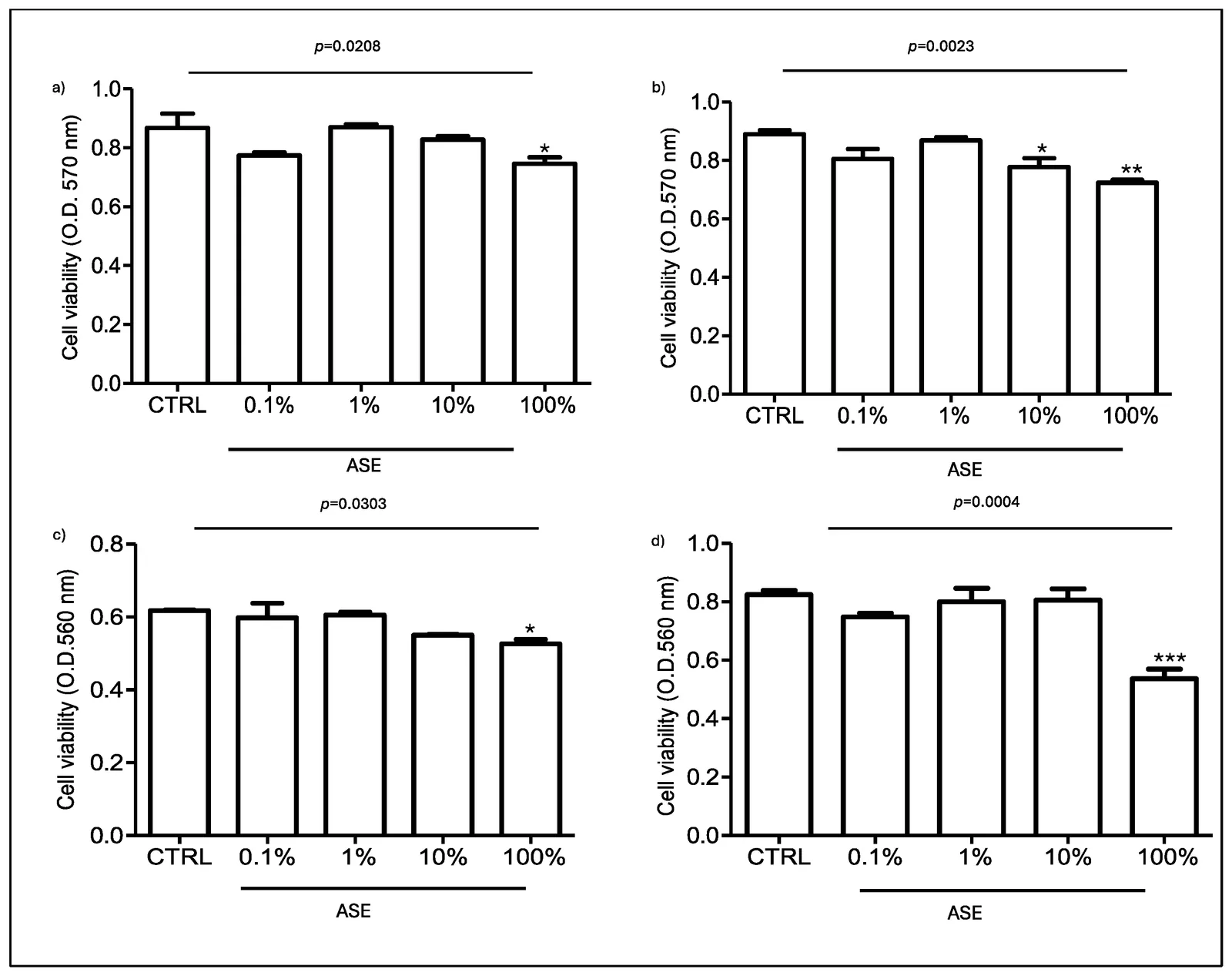
THP-1-derived macrophage viability. THP-1-derived macrophage viability was assessed by MTT (**a**,**b**) and NR (**c**,**d**) assays after 24 and 48 h of incubation with four different concentrations of ASE (100, 10, 1 and 0.1%). In the figure, (**a**,**c**) refer to 24 h, while (**b**,**d**) refer to 48 h. Data are expressed as mean ± SEM (n = 3). A one-way ANOVA was performed to assess overall differences between groups. Specific differences between each group and the control were determined using Tukey’s post hoc test. Asterisks indicate statistical significance levels compared to the control (* *p* < 0.05; ** *p* < 0.01; *** *p* < 0.001).

The results of the MTT assay showed that at 0.1% and 1% of ASE, cell viability remained close to control values at both time points. Exposure to 10% resulted in a moderate reduction in viability (4.61% at 24 h and 12.63% at 48 h). A further decrease was observed with 100% of ASE. The NR assay showed a similar trend, with cell viability reduced significantly only with ASE at 100% (Figure 7).

Overall, THP-1 cells appeared less sensitive to the treatment compared with the other cell models analyzed. Both the MTT and NR assay indicated a significant effect at 100%, more evident after 48 h of exposure.

## 4. Discussion

The utilization of efficient and safe methods for plant protection and stimulation without any harmful effect either on human health or on the environment is a challenge that has become increasingly urgent, guided by global economic crises and climate change. Thus, thanks to the presence of water-soluble bioactive compounds, garlic aqueous extract represents a promising natural alternative to synthetic pesticides, and unlike conventional pesticides, these extracts are selective and show no adverse effects on non-target organisms, including beneficial insects and soil microorganisms.

From a safety perspective, the risk assessment for agricultural workers associated with the use of garlic extract as a biopesticide has shown a favorable profile. Because it is a natural substance, rapidly biodegradable, and non-persistent in the environment, direct exposure during the preparation and application phases is considered preliminary evidence of low cytotoxicity under the tested conditions to human health.

The garlic chemical composition varies depending on extraction methods that determine bioactive concentration, territorial origin of garlic, and variety, and all of these factors collectively influence the effects of garlic.

Studies focusing on allicin, the major organosulfur compound derived from garlic, also demonstrated cytotoxic effects in non-cancerous mammalian cell lines. Human umbilical vein endothelial cells (HUVECs) and fibroblasts exposed to allicin showed reduced proliferation and viability in a concentration-dependent manner [45]. Several authors have investigated the cytotoxic effects of high concentrations (10%, 5%, 2.5%, and 1.25%) of garlic extract on treated tumor cell lines and showed different sensibilities, assessed on the base of IC_50_ values for the most sensitive and less sensitive cell line [46]; one relevant study evaluated the effects of aged garlic extract (AGE) and demonstrated the effects of proliferation and migration in normal cells, but not in cancer cells [47], suggesting that substantial activity depends on garlic variety and concentration.

Some evidence indicates that aqueous garlic extract may exert cytoprotective properties under oxidative stress conditions. For example, aqueous garlic extract was shown to reduce sodium arsenite-induced toxicity in HaCaT keratinocytes and normal human dermal fibroblasts, likely through antioxidant mechanisms [48].

The use of water for extraction has numerous advantages, such as positive environmental impact (water is non-toxic to humans and the environment and prevents pollution), the use of simple equipment, and the possibility of application in food and agriculture [32]. Thus, in this in vitro study we performed evaluation of aqueous garlic extract on multiple human cell lines representative of key exposure routes, including skin, respiratory, gastrointestinal, neuronal, and immune systems. To evaluate the cytocompatibility of ASE on different cell lines, we performed both the MTT and Neutral Red assays. In the MTT assay, the formazan product is impermeable to the cell membranes and therefore accumulates in healthy cells, detecting whether there is damage to the cell membrane; in the Neutral Red assay, living cells take up the Neutral Red, which is concentrated within the lysosomes and measures the viability of the cell. It must be kept in mind that each cell type often requires different concentrations to respond to signals, inducing variations in metabolic activity. Our results obtained by Neutral Red and MTT assays showed that higher concentrations (≥10%), which do not reflect typical exposure scenarios, induced a similar reduction in cell viability in all cell models. Additionally, no significant cytotoxic effects were observed at concentrations ≤1% at short and longer treatment periods. This response may be related to the activation of cellular stress pathways or sensitivity of cells to high concentrations of bioactive compounds. Given the high sensitivity of neuronal models to toxic compounds, the absence of neurotoxic effects at lower concentrations indicates a dose-dependent effect that should be considered when defining application protocols for agricultural use, supporting a favorable safety profile for potential exposure scenarios.

In summary, our result suggests that the aqueous extract does not impair cell viability under conditions relevant for chronic or acute occupational exposure, paving the way for the application of garlic extract for plant growth and development as well as protecting human health.

## 5. Conclusions

In conclusion, the aqueous garlic extract demonstrated a favorable cytocompatibility profile in several human cell models representing relevant exposure routes. No significant reduction in viability was observed at concentrations consistent with realistic exposure scenarios, while higher concentrations produced cell line-dependent effects, particularly in skin and immune cell models.

From a public health perspective, our preliminary results support the potential safe handling of the aqueous garlic extract for the implementation of safer and more sustainable pest management strategies, aimed at reducing reliance on synthetic pesticides and limiting occupational exposure of agricultural workers and promising improvements in nutraceutical and functional applications.

However, the results should be interpreted as an initial and preliminary in vitro safety assessment, requiring further characterization regarding repeated and long-term exposure, skin irritation and sensitization, inhalation toxicity, formulation stability, environmental fate, and in vivo exposure levels during preparation and application. Future research should focus on evaluating the expression of pro- and anti-inflammatory cytokines, the activation of key cellular signaling pathways (such as NF-κB and Nrf2), and the interaction with endogenous antioxidant defense systems. This knowledge could facilitate the development of standardized, safe, and effective formulations, intended for both sustainable agriculture and the production of nutraceuticals and functional agents capable of supporting the immune system. These aspects taken together are essential for defining safe use protocols, concentration limits, personal protection recommendations, and regulatory requirements.

## Data Availability

The data presented in this study are available on request from the corresponding author.
